# Effects of Two Kinds of Extracts of *Cistanche deserticola* on Intestinal Microbiota and Its Metabolism

**DOI:** 10.3390/foods11182897

**Published:** 2022-09-18

**Authors:** Yilin Li, Yalin Zhang, Xiaoming Su, Pengfei Zou, Xinyang Wang, Jie Chen, Xuan Zhu

**Affiliations:** 1 Key Laboratory of Healthy Mariculture for the East China Sea, Ministry of Agriculture and Rural Affairs, Ornamental Aquarium Engineering Research Centre in University of Fujian Province, Fisheries College, Jimei University, Xiamen 361021, China; 2 School of Food Science and Bioengineering, Zhejiang Gongshang University, Hangzhou 310018, China; 3 Weifang Elbe Health Food Co., Ltd., Weifang 261057, China

**Keywords:** *Cistanche deserticola*, alcohol extraction, aqueous extraction, gut microbiota, untargeted metabolomics

## Abstract

*Cistanche deserticola* belongs to the Liedang family. Known as “desert ginseng”, it has high medicinal value and plays important roles in endocrine regulation, neuroprotection, immune regulation, and other processes. Some studies have shown that single substances such as polysaccharides and phenylethanolside can affect intestinal microbiota, but few studies have studied the synergistic effect of various components in *Cistanche deserticola* extracts on intestinal microbiota. Therefore, in this study, through an in vitro digestion model (Changdao Moni, CDMN) combined with 16S rRNA gene amplification sequencing technology and untargeted metabolomics technology, it was found that the two extracts all had significant effects on the enteric cavity and mucosal flora. Both extracts inhibited *Bacteroides* in the intestinal cavity and *Parabacteroides* and *Ruminococcus 2* in the intestinal mucosa and promoted *Bifidobacterium* and *Prevotella* in the intestinal cavity and *Megasphaera* in the intestinal mucosa. The aqueous extract also inhibited *Phascolarctobacterium*. Both extracts also significantly increased the production of short-chain fatty acids, especially butyrate. The intake of extract had significant effects on the metabolic pathways related to amino acids and lipids. Indoles were upregulated by the aqueous extract but downregulated by the alcohol extract. In addition, the extract also had a significant effect on the hemolytic phosphorus esters. In conclusion, the two kinds of extracts have different effects on intestinal microbiota and its metabolism. This study provides guiding significance for the edibility and food development of *Cistanche deserticola*.

## 1. Introduction

*Cistanche deserticola* is a parasitic plant with extremely high medicinal value. It belongs to the genus *deserticola* in the Liedang family, which was originally included in “Shen Nong’s Chinese Materia Medica” and is known as “ginseng of the deserts”. As a parasitic plant, it is mainly parasitic on the roots of desert trees such as *Tamarix* species, *Haloxylon ammodendron*, and *Haloxylon persicum* [1]. These plants can survive in deserts such as the Gobi Desert and in saline–alkali land, where water resources are in short supply, with strong hardiness. *Cistanche deserticola* is currently the most common variety in the market, occupying the main position, and was officially recognized as the basic plant of “Chinese Pharmacopoeia” with *Cistanche tubulosa*. Modern pharmacological studies have shown that the main effects of *Cistanche deserticola* involve endocrine regulation, neuroprotection, immune regulation, antitumor, anti-inflammatory, and liver prevention processes [2]. Its main chemical components include phenylethanol glycosides, iridoids and their glycosides, lignan glycosides, oligosaccharides and oligosaccharides esters, polysaccharides, mannitol, betaine, and other components [1].

Many studies have reported that polysaccharides can regulate the structure and composition of intestinal microbiota, promote the growth of probiotics, and play an important role in maintaining intestinal health [3]. The results of bioactivity testing showed that the neutral polysaccharide obtained from *Cistanche deserticola* could significantly promote the growth of *Bacteroides* and of some probiotics, such as *Lactobacillus casei*, *Lactobacillus plantarum*, and *Lactobacillus reuteri*, which could help to maintain intestinal homeostasis [4]. Phenylethanol glycosides are the main active components in *Cistanche deserticola*, and intestinal microbiota also play an important role in its metabolism and pharmacology. Guo Yongli et al. performed animal experiments to confirm that phenylethanol glycosides can be metabolized into a series of small-molecule compounds, 3-HPP and HT, under the action of intestinal microbiota and play a protective role in the liver [5]. Another study found that phenylethanol glycosides could regulate the structure of intestinal microbiota in rats, reduce the relative abundance of *Firmicutes* and *Bacteroidetes*, and change the functional pathway of intestinal microbiota [6]. In conclusion, intestinal microbiota may play an important role in the metabolism of *Cistanche deserticola* after its ingestion.

However, the current reports usually study the pharmacological action or metabolism of a single component of *Cistanche deserticola* through mouse experiments, and few study the synergistic effect of *Cistanche deserticola* extracts on intestinal microbiota. The common way to eat *Cistanche deserticola* is to stew it, make wine, make tea, and related methods. In traditional Chinese medicine, the main method of usage is to decoct in water. Therefore, this study established an in vitro digestion model (Changdao Moni, CDMN) to explore the effects of aqueous and alcohol extracts of *Cistanche deserticola* on intestinal microbiota and metabolism and to evaluate their potential effects on the human body, which will provide a theoretical basis for the development and consumption of *Cistanche deserticola*-related foods to expand the application scope of *Cistanche deserticola*. The results of this study will be of great significance to the development of the *Cistanche deserticola* industry.

## 2. Materials and Methods

### 2.1. Preparation of Cistanche deserticola Extracts

#### 2.1.1. Preparation of an Ethanol Extract of *Cistanche deserticola*

A total of 25 g of *Cistanche deserticola* sample was weighed, cut into pieces, and extracted twice with 80% ethanol at 70 °C for 2 h, and the solid–liquid ratio was 1:10 (*w*:*v*). The residue was filtered with gauze, and the filtrate was combined. The filtrate was concentrated to a certain concentration by a rotary evaporator, lyophilized into powder, and then redissolved with water to 1 g (crude drug concentration)/mL.

#### 2.1.2. Preparation of an Aqueous Extract of *Cistanche deserticola*

Twenty-five grams of *Cistanche deserticola* was weighed, cut into small pieces, and extracted by boiling 10 times the amount of water 3 times, and each extraction time was 1 h. The residue was filtered by gauze, and the filtrate was combined 3 times and concentrated to 1 g (crude drug concentration)/mL.

### 2.2. In Vitro Gastrointestinal Digestion of Extracts

#### 2.2.1. In Vitro Oral Digestion

Five milliliters of each extract of *Cistanche deserticola* was added to 3.5 mL of SSF (simulated salivary fluid) electrolyte reserve solution, mixed and minced. The SSF solution consisted of 15.1 mmol/L KCl, 3.7 mmol/L KH_2_PO_4_, 13.6 mmol/L NaHCO_3_, 0.15 mmol/L MgCl_2_(H_2_O)_6_, and 0.06 mmol/L (NH_4_)_2_CO_3_. A 0.5 mL saliva α-amylase solution consisting of SSF electrolyte reserve solution was added (to achieve a final activity of 75 U/mL in the digestive system), followed by 25 μL of 0.3 mol/L CaCl_2_ and 975 μL of distilled water, and the mixture was thoroughly mixed. Because the oral digestion time is short, it is recommended to preheat all reagents to 37 °C in advance to maximize enzyme activity. The final mixture was digested in a 37 °C water bath oscillator for 2 min [7].

#### 2.2.2. In Vitro Gastric Digestion and Small Intestinal Digestion

A 7.5 mL SGF (simulated gastric fluid) electrolyte reserve solution was added to the 10 mL oral digestive fluid obtained above. The SGF solution consisted of 6.9 mmol/L KCl, 0.9 mmol/L KH_2_PO_4_, 25 mmol/L NaHCO_3_, 47.2 mmol/L NaCl, 0.1 mmol/L MgCl_2_(H_2_O)_6_, and 0.5 mmol/L (NH_4_)_2_CO_3_. Then, 1.6 mL pepsin solution was dissolved in SGF electrolyte reserve solution (to make its final activity in the digestive system reach 2000 U/mL enzymatic activity), and then 5 μL of 0.3 mol/L CaCl_2_ was added, followed by an appropriate amount of 1 mol/L HCl to adjust the pH to 3.0. Distilled water was added until the system reached 20 mL, and the mixture was thoroughly mixed. The digestion was carried out at 37 °C for 2 h by shaking [7].

To the 20 mL gastric digestive fluid obtained above, 11 mL of SIF (simulated intestinal fluid) was added, along with 5 mL of trypsin solution dissolved from the SIF electrolyte reserve solution, bringing the final system to 100 U/mL enzymatic activity (based on trypsin activity). The SIF solution consisted of 6.8 mmol/L KCl, 0.8 mmol/L KH_2_PO_4_, 85 mmol/L NaHCO_3_, 38.4 mmol/L NaCl, and 0.33 mmol/L MgCl_2_(H_2_O)_6_. Then, 2.5 mL of bile was added, 40 μL of 0.3 mol/L CaCl_2_ was added, the pH was adjusted to 7 with 1 mol/L NaOH, the volume was fixed to 40 mL with water, and the digestion was carried out by shaking in a water bath at 37 °C for 2 h [7].

### 2.3. Determination of Chemical Composition of Extracts and Their Digested Products

#### 2.3.1. Determination of Total Free Amino Acid Content

One milliliter of sample solution was centrifuged at 4000 r/min for 5 min, and the supernatant was diluted to an appropriate multiple. Three milliliters of diluent were added to 1.0 mL pH 5.0 sodium acetate buffer solution and 2.0 mL 2.0% ninhydrin solution, and heated in a water bath at 100 °C for 19 min. After cooling for 10 min, the absorbance was measured at 568 nm and taken into the standard curve for calculation.

#### 2.3.2. Determination of Reducing Sugar Content

A total of 1 mL sample was centrifuged at 4000 r/min for 5 min, and the supernatant was diluted to an appropriate multiple. A 50 μL sample dilution was taken to a clean centrifuge tube, then 100 μL DNS detection solution was added and carefully mixed. It was accurately boiled in a boiling water bath for 5 min, cooled to room temperature with tap water, and then supplemented with 250 μL distilled water. The absorbance was measured at 540 nm and calculated by bringing it into the standard curve.

#### 2.3.3. Determination of Phenylethanol Glycosides Content

A total of 2 mL of sample was absorbed into the centrifuge tube and centrifuged at 4000 r/min for 5 min, then 1 mL of supernatant was taken and mixed with 3 mL water-saturated n-butanol by shaking, centrifuged at 3000 r/min for 15 min, dried with nitrogen at 37 °C, and redissolved with appropriate amount of distilled water. Two milliliters of sample solution was taken to 25 mL volumetric flask, then 1 mL of 5% sodium nitrite solution was added, shaken well, and left to stand for 6 min, then 1 mL of 10% aluminum nitrate solution was added, shaken well, and left to stand for 6 min, then 10 mL of 10% sodium hydroxide solution was added and diluted with distilled water to the scale line. The absorbance value was measured at 510 nm, and the content of phenylethanol glycosides in the extract was calculated by using the standard curve.

### 2.4. In Vitro Colonic Fermentation of Extracts

#### 2.4.1. Collection and Handling of Fecal Microorganisms

Stool samples were collected from more than 3 healthy young people aged 20–25 years who had no intestinal diseases, including inflammatory enteritis and irritable bowel syndrome, and had not used antibiotics within six months as the source of intestinal microbiota. The feces were mixed with sterile PBS buffer at a ratio of 1:8, thoroughly stirred and suspended, and the solid particles were filtered out with three layers of sterile gauze. The filtration was repeated three times, and nitrogen was immediately injected to remove the oxygen in the liquid, after which the filtrate was then placed in an anaerobic environment for use.

#### 2.4.2. Preparation of Mucosal Pellets

Two grams of agar was weighed and dissolved in 100 mL of distilled water, heated, and stirred until dissolved. When the agar solution was clear and transparent, the heating was stopped and cooled to approximately 60 °C. Then, 0.5 g of mucin was added and dissolved while hot, and the pH of the solution was adjusted to approximately 6.8. The solution was then poured into a spherical abrasive tool that was sterilized by ultraviolet light for 30 min. After cooling, a mucosal gel ball with a diameter of 7–8 mm was obtained. This process was carried out on an ultraclean workbench.

#### 2.4.3. In Vitro Colon Simulation

Each fermenter was supplemented with 270 mL intestinal medium, 30 mL fecal bacterial solution, and 15 mucosal pellets. The basal medium contained the following constituents (g/L) in distilled water: peptone (3.0), corn starch (8.0), yeast extract (4.5), tryptone (3.0), mucin (0.5), L-cysteine hydrochloride (0.8), bile No. 3 (0.4), heme (0.05), sodium chloride (4.5), Tween-80 (1.0), potassium chloride (2.5), potassium dihydrogen phosphate (0.4), magnesium chloride hexahydrate (4.5), calcium chloride hexahydrate (0.2), and 2.0 mL stock solution (3.0 g/L magnesium sulfate heptahydrate, 0.1 g/L ferrous sulfate heptahydrate, 0.1 g/L calcium chloride dihydrate, 0.32 g/L manganese chloride tetrahydrate, 0.18 g/L cobalt sulfate heptahydrate, 0.01 g/L copper sulfate pentahydrate, 0.18 g/L zinc sulfate heptahydrate, and 0.092 g/L nickel chloride hexahydrate) [8]. By setting the parameters of the CDMN intestinal fermentation system, 0.5 mol/L NaOH solution and 0.5 mol/L HCl were automatically added to adjust the fermentation pH to approximately 5.8, the temperature was constant at 37 °C, and the stirring speed was set at 150 r/min, which simulated the fermentation environment of the human ascending colon. Nitrogen was fed to each fermenter early, mid, and late daily to maintain an anaerobic environment. The medium was supplemented and the waste discharged at a certain flow rate every day to ensure that the supply and discharge after 24 h were both 100 mL to simulate human ingestion and digestion. Every day, the mucosal balls in the tank were replaced with five new balls to simulate the regeneration of the intestinal mucosa.

Colonic fermentation is divided into two stages. The first stage is the stable stage. After fecal bacterial solution is added, the fermentation lasts for approximately 7 days, which makes the microbial structure in the fermenter stable. In the second stage, samples are added to understand the influence of samples on the structure of fecal flora. In this study, the added samples were the intestinal digestive fluid samples of the aqueous and alcohol extracts of *Cistanche deserticola*, and the concentration of the extract in the final system was 1.7%. *Cistanche deserticola* was digested in the stomach and small intestine according to the above method and added to the fermenter regularly every day, and the fermentation was stopped after 7 days. The fermentation broth and mucosal pellets from each fermenter on days 0–7 of the second stage were sampled daily, and the samples were stored at −20 °C [9].

### 2.5. Fermentation Gas Determination

Every day, 10 mL of fermentation liquid from each tank was measured in a glass test tube, sealed with a sealing film, and placed in an incubator at 37 °C for 30 min to volatilize the gas in the fermentation liquid. The changes in the main components of the gas were measured with an electronic nose. The parameters of the Smart Nose software were as follows: cleaning time—120 s; measuring time—75 s; washing gas—standard air.

### 2.6. 16S rDNA Sequencing

Bacterial genomes were extracted from the fermentation broth and mucosal pellets before adding samples and from the fermentation broth and mucosal pellets on the 2nd, 4th, and 6th days after adding samples according to the instructions of the TIANGEN Bacterial DNA Extraction Kit. DNA concentration and purity were determined by a NanoDrop instrument, and qualified DNA samples were sent to LC-Bio Technology Co., Ltd. (Hang Zhou, Zhejiang Province, China) for 16S rDNA gene sequencing analysis.

### 2.7. Short-Chain Fatty Acid Extraction and Determination

One milliliter of fermentation broth was transferred to a clean centrifuge tube and centrifuged at 10,000 r/min for 10 min at 4 °C, and the supernatant was retained after the precipitate was discarded. Then, 100 μL concentrated hydrochloric acid and 5 mL diethyl ether were added and mixed well, and the supernatant was collected by static extraction for 20 min at room temperature, followed by centrifugation at 5000 r/min for 10 min at 4 °C. One milliliter of 1 M sodium hydroxide solution was added, and the solution was mixed well, extracted at room temperature for 20 min, and centrifuged at 5000 r/min for 10 min at 4 °C. Then, 400 μL of the lower liquid was collected, and 100 μL of concentrated hydrochloric acid was added and mixed. Finally, the obtained sample was filtered through a 0.22 μm filter membrane.

Detection conditions: column—ZORBAX SB-Aq (4.6 × 250 mm 5-micron); mobile phase—0.025% phosphate aqueous solution (pH = 2.8), acetonitrile = 95:5; flow rate—1.0 mL/min; injection volume—20 μL; detection wavelength—210 nm; column temperature—30 °C [10].

### 2.8. Untargeted Metabolome Detection

All samples were acquired by the LC-MS system following the machine order. First, all chromatographic separations were performed using an ultraperformance liquid chromatography (UPLC) system (SCIEX, Macclesfield, UK). An ACQUITY UPLC T3 column (100 mm × 2.1 mm, 1.8 µm, Waters, UK) was used for reversed-phase separation. The column oven was maintained at 35 °C. The flow rate was 0.4 mL/min, and the mobile phase consisted of solvent A (water, 0.1% formic acid) and solvent B (acetonitrile, 0.1% formic acid). Gradient elution conditions were set as follows: 0~0.5 min, 5% B; 0.5~7 min, 5% to 100% B; 7~8 min, 100% B; 8~8.1 min, 100% to 5% B; 8.1~10 min, 5% B. The injection volume for each sample was 4 µL.

A high-resolution TripleTOF 5600 Plus (SCIEX, UK) tandem mass spectrometer was used to detect metabolites eluted from the column. Q-TOF was operated in both positive and negative ion modes. The curtain gas was set to 30 PSI, ion source gas1 was set to 60 PSI, ion source gas2 was set to 60 PSI, and an interface heater temperature was 650 °C. For positive ion mode, the ion spray floating voltage was set at 5000 V. For negative ion mode, the ion spray floating voltage was set at −4500 V. The mass spectrometry data were acquired in IDA mode. The TOF mass range was from 60 to 1200 Da. The survey scans were acquired in 150 ms, and as many as 12 product ion scans were collected if exceeding a threshold of 100 counts per second (counts/s) and with a 1+ charge-state. The total cycle time was fixed to 0.56 s.

Four time bins were summed for each scan at a pulser frequency value of 11 kHz through monitoring of the 40 GHz multichannel TDC detector with four-anode/channel detection. Dynamic exclusion was set for 4 s. During the acquisition, the mass accuracy was calibrated every 20 samples. Furthermore, to evaluate the stability of the LC-MS during the whole acquisition, a quality control sample (pool of all samples) was acquired after every 10 samples.

## 3. Results

### 3.1. Overview of Chemical Composition of Extracts and Their Digested Products

In this study, we determined three main chemical constituents of *Cistanche deserticola* extracts and their digested products. According to Table 1, the main difference between the aqueous extract and alcohol extract was phenylethanol glycosides; the content of phenylethanol glycosides in the alcohol extract was significantly higher than that in water extract (*p* < 0.05). However, there was no significant difference in the content of reducing sugar and total free amino acid between the aqueous extract and alcohol extract (*p* > 0.05). After gastric digestion, the main difference was in the content of reducing sugar, and the content of reducing sugar in the alcohol extract group was significantly higher than that in the aqueous extract group (*p* < 0.05). There was also no significant difference in the content of phenylethanol glycosides and total free amino acid between the aqueous extract and alcohol extract (*p* > 0.05). After small intestinal digestion, the result was same as that after gastric digestion.

### 3.2. Effect of Extracts on the Alpha Diversity of Intestinal Microbiota

**Figure 1:**
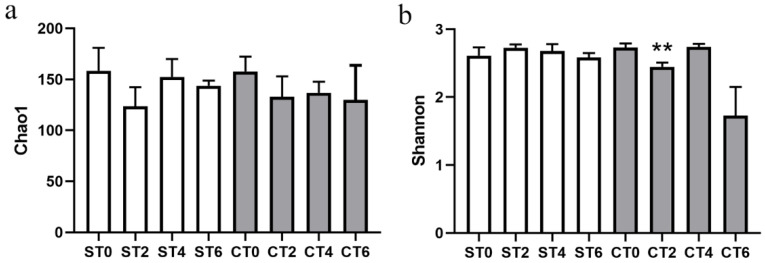
Histogram analysis of the alpha diversity of the intestinal microbiota. (**a**) Chao1 index bar chart; (**b**) Shannon index bar chart (ST—aqueous extract group; CT—alcohol extract group; 0—no sample added; 2, 4, 6—denote fermentation days after sample addition; ** *p* < 0.01, with no sample added as the control).

The Chao1 index reflects community richness, while the Shannon index reflects community richness and evenness. Generally, the larger the Chao1 index is, the higher the richness of the community. Similarly, the higher the Shannon index is, the higher the community diversity. The two groups of samples had no significant effect on the Chao1 index, indicating that they had no effect on the types of intestinal microbiota. On the second day after the intake of the ethanol extract of *Cistanche deserticola*, the Shannon index was significantly decreased, which affected the species diversity, but the Shannon index fluctuated in the subsequent intake but had no significant effect (Figure 1). Overall, the continuous intake of the two groups of samples had no significant effect on the alpha diversity of the gut microbiota.

### 3.3. Effect of Extracts on the Beta Diversity of the Microbiota

Beta diversity is used to calculate the distance between samples by using the evolutionary relationship and abundance information of each sample sequence to reflect whether there are significant microbial community differences among samples (groups). Principal component analysis (PCA) is a visualization method to study the similarity or difference of data, through which the differences between individuals or groups can be observed.

**Figure 2:**
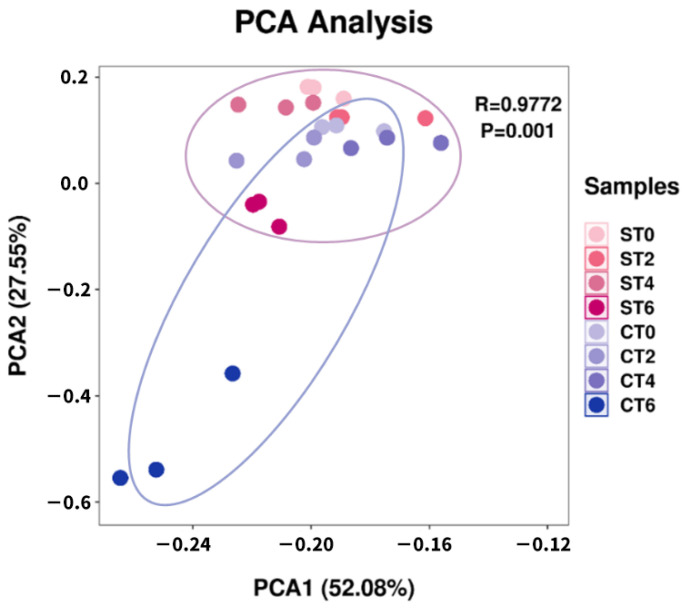
Principal component analysis of intestinal microbiota (ST—aqueous extract group; CT—alcohol extract group; 0—no sample added; 2, 4, 6 denote fermentation days after sample addition).

As shown in Figure 2, in the two-dimensional graph of the first principal component (52.08%) and the second principal component (27.55%) of the PCA, the distance between samples represents the similarity of microbial communities in samples. The closer the distance is, the higher the similarity. With the continuous intake of samples, the intestinal microorganisms treated with aqueous extract and alcohol extract gradually showed differences, and on the sixth day, the difference between the two groups reached the maximum, and the difference within the group also reached the maximum.

### 3.4. Effects of the Two Extracts on the Abundance of Phyla in Intestinal Microbiota

**Figure 3:**
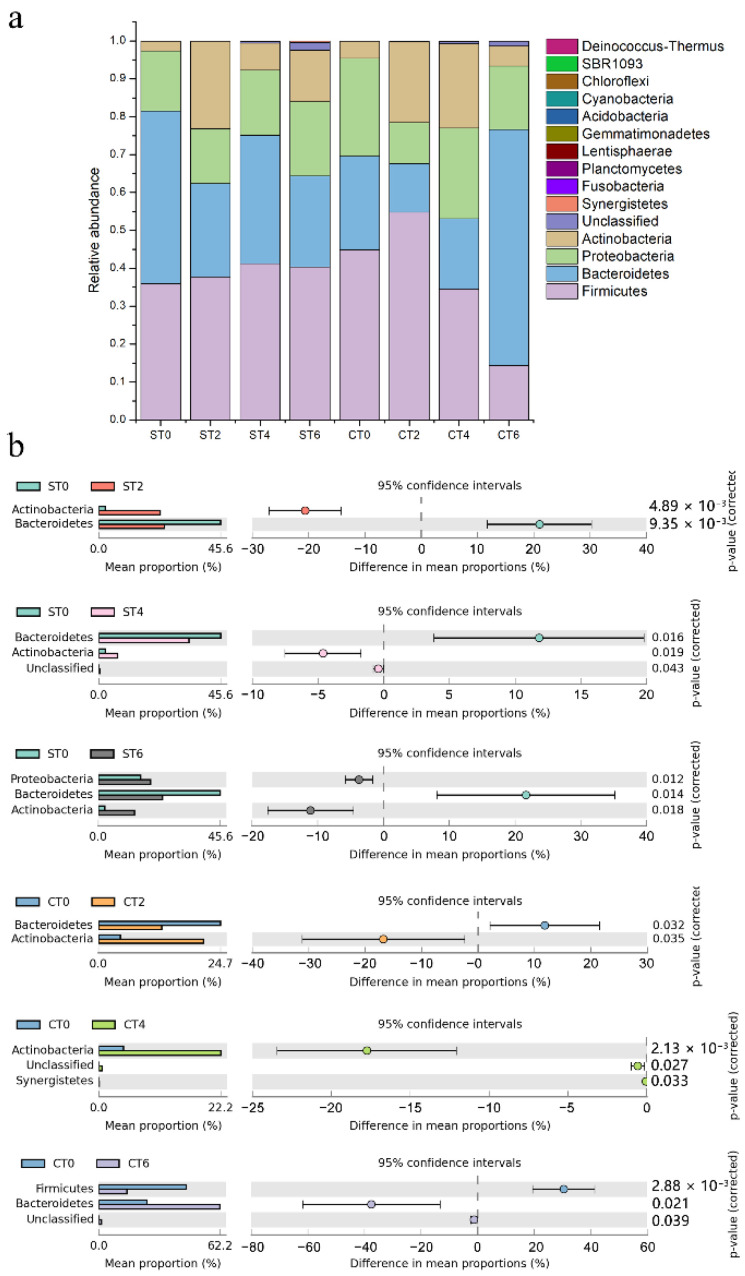
Bar and differential analysis charts of the relative abundance of phyla in the intestinal lumen microflora. (**a**) Bar chart of the relative abundance of microbial phyla in the colonic lumen. (**b**) Difference analysis chart between groups (ST—aqueous extract group; CT—alcohol extract group; 0—no sample added; 2, 4, 6 denote fermentation days after sample addition).

Specific gut microbiota were analyzed at the phylum level (Figure 3) to evaluate the effect of *Cistanche deserticola* extract on specific gut microbiota. According to the Figure 3, *Firmicutes* accounted for the largest proportions of approximately 14–55%, followed by *Bacteroidetes*, *Proteobacteria*, and *Actinobacteria*, among others, which constitute the intestinal microbiota of the human body. According to the significance analysis of STAMP, the intervention of aqueous extract significantly inhibited *Bacteroidetes*, and after calculating the Firm/Bacter ratio, it was found that compared with the control group, the intake of aqueous extract of *Cistanche deserticola* increased the ratio. Over time, the ratio increased from 0.79 to 1.53 and from 1.22 to 1.68.

However, in the alcohol extract group, the intestinal microbiota showed another change. On the second day after the alcohol extract intervention, *Bacteroidetes* were significantly inhibited, and *Actinobacteria* were significantly promoted. On the fourth day, *Actinobacteria* and *Synergistetes* were significantly promoted. On the sixth day after continuous intervention, *Firmicutes* were significantly inhibited, while *Bacteroidetes* were significantly promoted. In conclusion, the influence of alcohol extraction on *Bacteroidetes* changed from inhibition to promotion and could significantly inhibit *Firmicutes* after continuous intake. According to the calculation of the Firm/Bacter ratio, the sample intervention increased the ratio from 1.82 to 4.26, after which it returned to 1.87 and finally decreased to 0.23 on the sixth day.

### 3.5. Effects of Two Extracts on the Genus Level of Intestinal Microbiota

**Figure 4:**
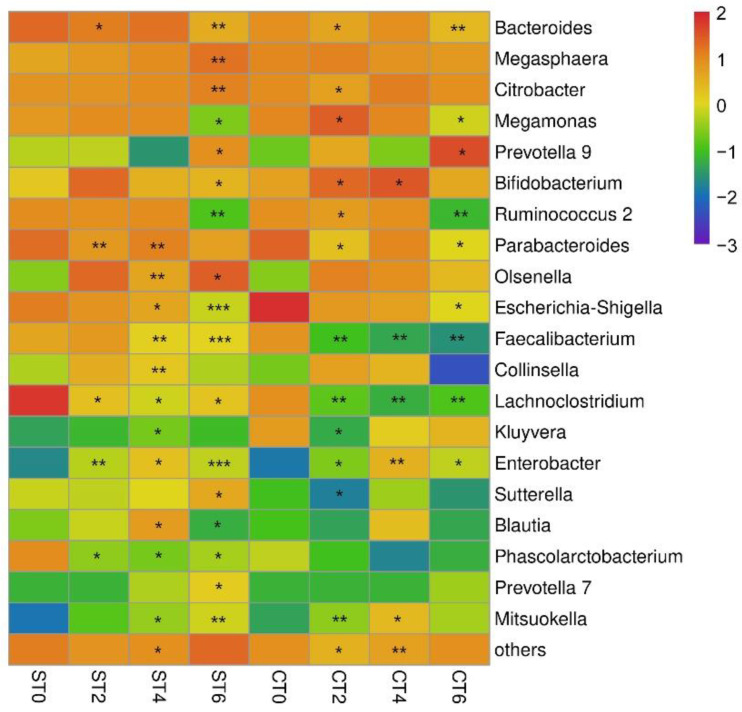
Heatmap of the relative abundance of bacterial genera in the intestinal lumen microbiota (ST—aqueous extract group; CT—alcohol extract group; 0—no sample added; 2, 4, 6 denote fermentation days after sample addition; with no sample added as the control, * *p* < 0.05, ** *p* < 0.01, *** *p* < 0.001).

At the genus level (Figure 4), this study focused on the relative abundances of the top 20 genera. The microorganisms in the intestinal lumen mainly included *Bacteroides*, *Megasphaera*, *Citrobacter*, *Ruminococcus*, and other bacteria.

The main difference between the two extracts after intake was that the aqueous extract needed long-term intake to promote the growth of beneficial bacteria *Bifidobacterium*, while the alcohol extract promoted the growth of *Bifidobacterium* at the early stage of intake but restored it to the initial level after long-term intake. Although the relative abundance of *Prevotella* increased significantly in both groups on the sixth day, the alcohol extract increased it to more than 50% in the alcohol extract group, indicating that the alcohol extract could increase the relative abundance of *Prevotella* more than the aqueous extract of *Cistanche deserticola*. In addition, the relative abundance of *Blautia* fluctuated during the intake of aqueous extract, while there was no significant change in the alcohol extract group. Moreover, the aqueous extract group significantly inhibited *Phascolarctobacterium*. Both groups had significant and similar inhibitory effects on *Bacteroides*, *Faecalibacterium*, and *Escherichia-Shigella*, but had significant promoting effects on *Enterobacter*.

### 3.6. Effects of Extracts on Intestinal Mucosa Microbiota

**Figure 5:**
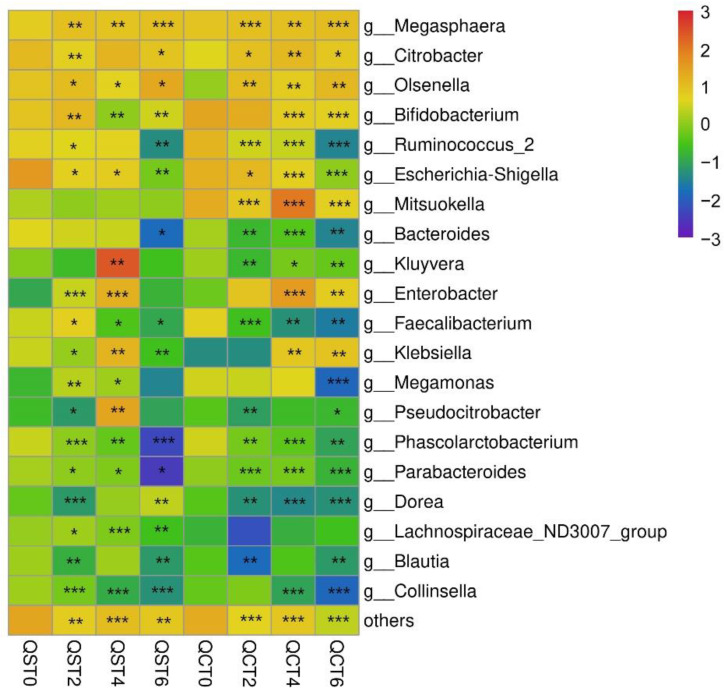
Heatmap of the relative abundances of bacterial genera in the intestinal mucosal microbiota (ST—aqueous extract group; CT—alcohol extract group; 0—no sample added; 2, 4, 6 denote fermentation days after sample addition; with no sample added as the control, * *p* < 0.05, ** *p* < 0.01, *** *p* < 0.001).

According to Figure 5, the main bacterial genera in mucosa were *Megasphaera*, *Citrobacter*, *Olsenella*, *Bifidobacterium*, and other microorganisms.

The effects of the aqueous extract and alcohol extract on intestinal mucosa microbiota were different. The aqueous extract had significant effects on *Lachnospiraceae*, *Bifidobacterium*, and *Faecalibacterium*, from a significant promotion to a significant inhibition, while the alcohol extract had no significant effect on *Lachnospiraceae* but had an inhibitory effect on both *Bifidobacterium* and *Faecalibacterium*. In addition, the two groups of samples also had different effects on *Dorea*, *Klebsiella*, *Bacteroides*, and *Olsenella*. The effect of the aqueous extract on *Dorea* changed from significant inhibition to significant promotion, while the effect of the alcohol extract on *Dorea* was always significant inhibition. The effect of aqueous extract on *Klebsiella* fluctuated, while the long-term intake of alcohol extract significantly promoted the effect. Whereas alcohol extract inhibited the relative abundance of *Bacteroides* immediately after intervention, aqueous extract had a significant inhibitory effect on *Bacteroides* only after long-term intervention. While *Olsenella* was in a fluctuating state when the aqueous extract was applied, it was in a significantly promoted state when the alcohol extract was applied. Similarly, both groups of samples significantly promoted *Megasphaera* and *Enterobacter* in mucosa, while *Parabacteroides*, *Ruminococcus 2*, *Escherichia-Shigella*, and *Collinsella* were inhibited.

### 3.7. Gas Production by Gut Microbes

**Figure 6:**
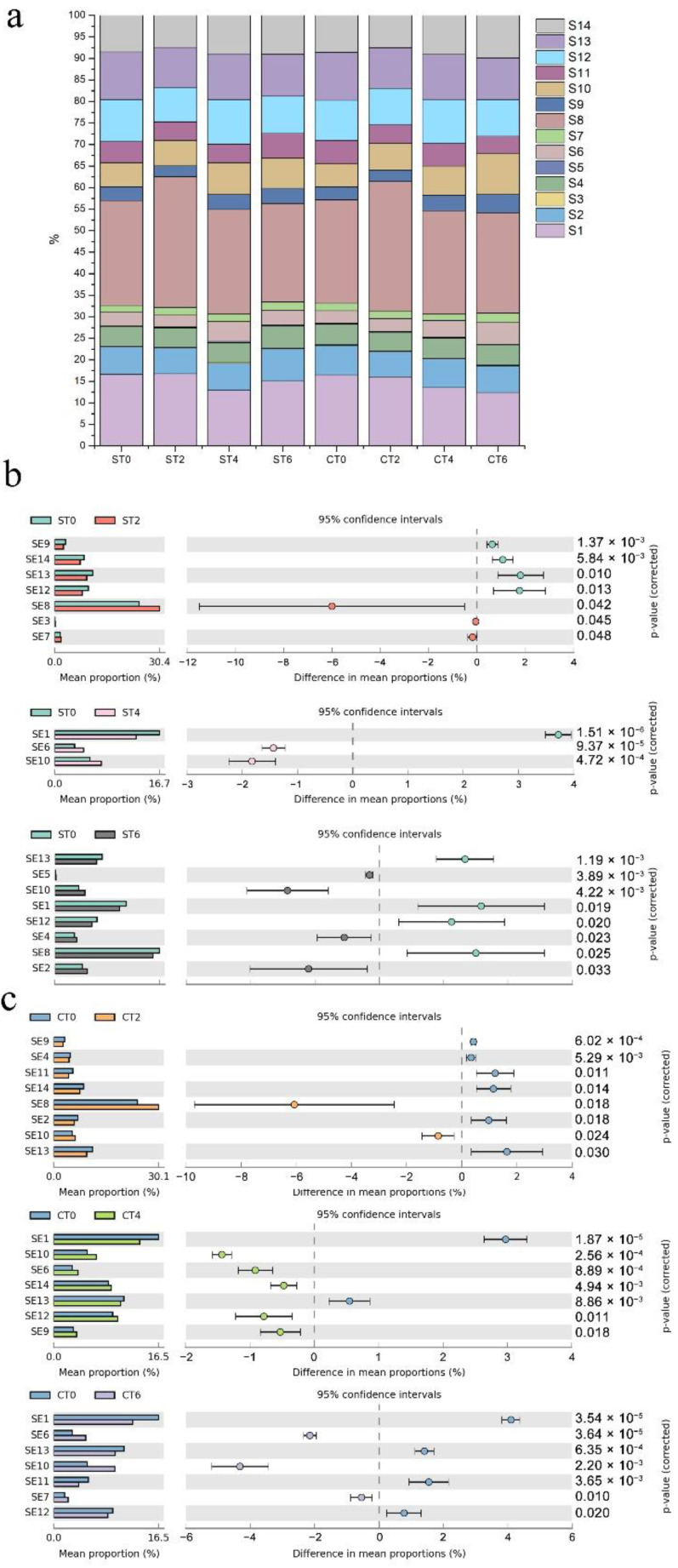
Histogram of the proportion of gas produced by intestinal microorganisms and the difference between groups. (**a**) Bar chart of gas composition; (**b**) differences between groups of aqueous extract; (**c**) differences between groups of alcohol extract (0—no sample added; 2, 4, 6 denote fermentation days after sample addition). (S1—ammonia and amines; S2—hydrogen sulfide, sulfide; S3—hydrogen; S4—alcohol, organic solvents; S5—alcohols, ketones, aldehydes, aromatic compounds; S6—methane, biogas, natural gas; S7—combustible gas; S8—VOC; S9—liquefied gas, natural gas, gas; S10—liquefied gas, flammable gas; S11—alkanes, alcohol, natural gas, smog; S12—alcohol, organic solvents; S13—smoke, cooking stink; S14—methane gas reservoir).

Ammonia, hydrogen sulfide, hydrogen, and methane are the main components of intestinal gas. According to Figure 6, the intake of aqueous extract increases the proportions of hydrogen, methane, hydrogen sulfide, and other gases, while significantly reducing the proportion of ammonia. Alcohol extract intake mainly significantly reduced the proportions of methane and hydrogen sulfide, while sustained intake was found to significantly increase the proportion of methane and significantly reduce the proportion of ammonia.

### 3.8. Effects of the Two Extracts on Intestinal Lumen Short-Chain Fatty Acids (SCFAs)

When using this method to detect short-chain fatty acids, the limits of detection of acetic acid, propionic acid, butyric acid, and valeric acid were 0.0025 mL/L, 0.005 mL/L, 0.01 mL/L, and 0.05 mL/L, respectively. The limits of quantitation of acetic acid, propionic acid, butyric acid, and valeric acid were 0.01 mL/L, 0.015 mL/L, 0.04 mL/L, and 0.1 mL/L, respectively.

**Figure 7:**
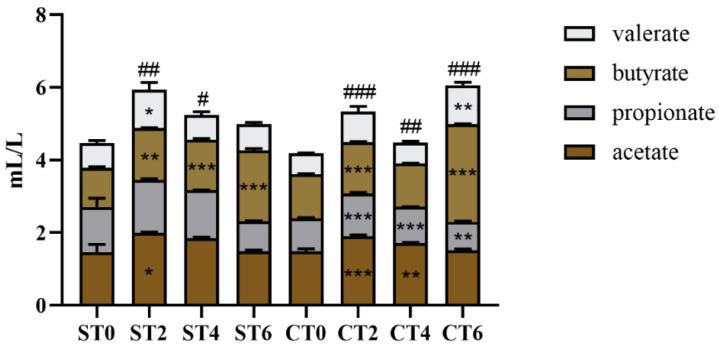
Content of SCFA in intestinal lumen (ST—aqueous extraction group, CT—alcohol extraction group; Comparison of SCFA between groups: * *p* < 0.05 ** *p* < 0.01 *** *p* < 0.001; Total acid contrast: # *p* < 0.05, ## *p* < 0.01, ### *p* < 0.001, compared with Day 0 for significant differences; 0—no sample added; 2, 4, 6—denote fermentation days after sample addition).

As shown in Figure 7, intake of the aqueous extract of *Cistanche deserticola* significantly increased the concentration of total SCFA mainly because it promoted the concentration of butyrate (*p* < 0.01 or *p* < 0.001), while acetate and valerate concentrations also increased significantly on the second day of sample intervention (*p* < 0.05). Although propionate was not statistically significant, it also tended to increase on the second day of aqueous extract intake. These results indicated that the intake of the aqueous extract of *Cistanche deserticola* affected the production of short-chain fatty acids, especially butyric acid, in the intestinal microbiota. Similarly, the intervention of alcohol extract resulted in the content changes of four SCFAs, among which butyrate significantly increased, propionic acid changed from significantly increased to significantly decreased after long-term intervention of alcohol extract, acetic acid changed from significantly increased to no significant change, and valerate content increased significantly on the sixth day.

In conclusion, we found that both extracts of *Cistanche deserticola* could promote the production of SCFAs, especially butyric acid. However, the ethanol extract of *Cistanche deserticola* inhibited the production of propionic acid and promoted the production of valeric acid after long-term intake.

### 3.9. Effects of the Two Extracts on Short-Chain Fatty Acids of the Intestinal Mucosa

**Figure 8:**
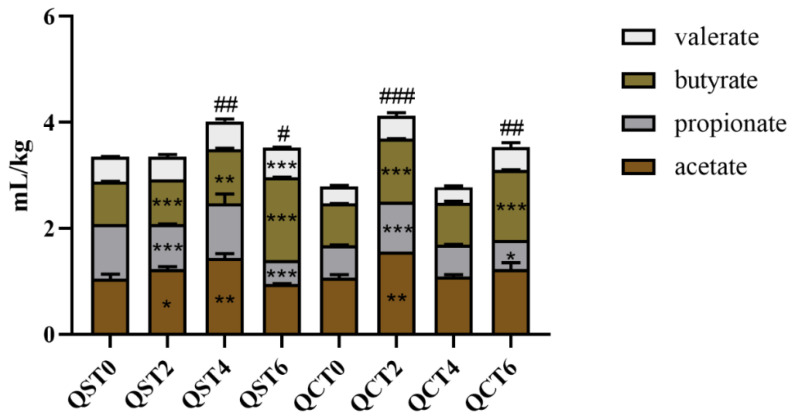
Content of SCFA in intestinal mucosa (QST—aqueous extraction group, QCT—alcohol extraction group. Comparison of SCFA between groups: * *p* < 0.05 ** *p* < 0.01 *** *p* < 0.001. Total acid contrast: # *p* < 0.05, ## *p* < 0.01, ### *p* < 0.001, compared with Day 0 for significant differences. 0—no sample added; 2, 4, 6 denote fermentation days after sample addition).

As shown in Figure 8, after aqueous extract intake, SCFA levels in the mucosa increased significantly on Days 4 and 6, mainly due to a significant increase in butyrate, similar to the results in the intestinal lumen. However, propionic acid in the mucosa decreased significantly on the 2nd and 4th days, while valeric acid showed a trend of increasing significantly.

However, after alcohol extract intervention, SCFAs in the mucosa increased significantly on the 2nd and 6th days, valerate did not change significantly, butyrate also showed a significant increasing trend, and propionate significantly decreased after a significant increase. The long-term intake of both extracts led to reductions in propionate in the intestinal mucosa but promoted the production of butyrate.

### 3.10. Analysis of Differential Metabolites of Intestinal Microbiota

In the fermentation broth samples, 5874 and 6590 variables were detected in the positive and negative ion modes, respectively. After water extract intervention, 2357 and 2413 variables were detected in positive and negative ion modes, respectively. After alcohol extract intervention, 2824 and 3027 variables were detected in positive and negative ion modes, respectively.

**Figure 9:**
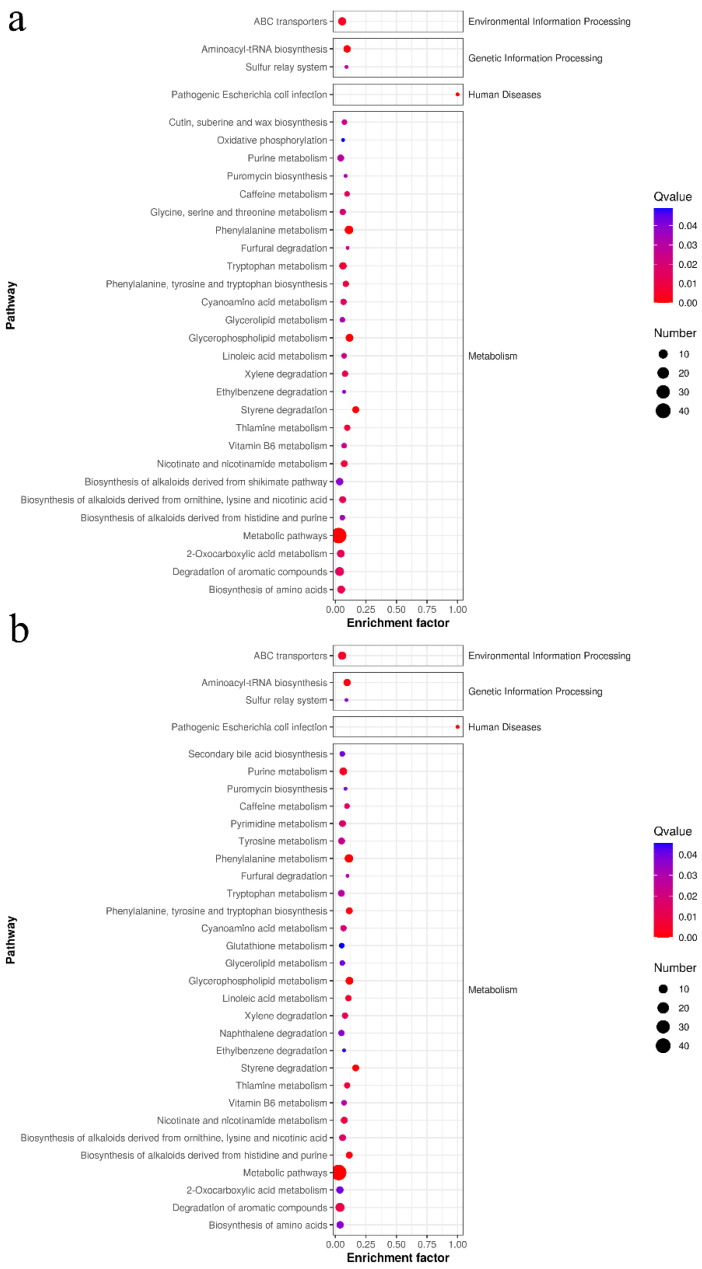
Bubble diagram of the different metabolic pathways in the aqueous extraction group (**a**) and the alcohol extraction group (**b**).

According to the detected differential metabolites between groups, metabolites that can be enriched in metabolic pathways in the KEGG database were selected for significance and visualization analyses of metabolic pathways. Ggplot2 was used to display the KEGG enrichment analysis results in a scatter plot (Figure 9). The enrichment factor indicates the number of differential metabolites in the KEGG/the total number of metabolites in the KEGG. The smaller the P value is, the higher the enrichment degree of KEGG. In general, sample intervention could significantly affect the related pathways of amino acid metabolism, lipid metabolism, and cofactor and vitamin metabolism of the gut microbiota. As shown in Figure 9, the intake of aqueous extract mainly significantly affected 31 tertiary metabolic pathways and the intervention of alcohol extract significantly affected 32 metabolic pathways, while both affected 27 metabolic pathways. These differences included biosynthesis of amino acids, degradation of aromatic compounds, and caffeine metabolism. In addition, the aqueous extract also significantly affected glycine, serine, and threonine metabolism and biosynthesis of alkaloids derived from the shikimate pathway and other metabolic pathways, while the alcohol extract group had significant effects on tyrosine metabolism, secondary bile acid biosynthesis, glutathione metabolism, and other metabolic pathways. Most of these significantly different metabolic pathways belong to secondary metabolic pathways of amino acid and lipid metabolism. Therefore, amino acid and lipid metabolites were mainly analyzed in the follow-up.

### 3.11. Analysis of Metabolites Related to the Amino Acid Metabolism Pathways of Intestinal Microbiota

**Figure 10:**
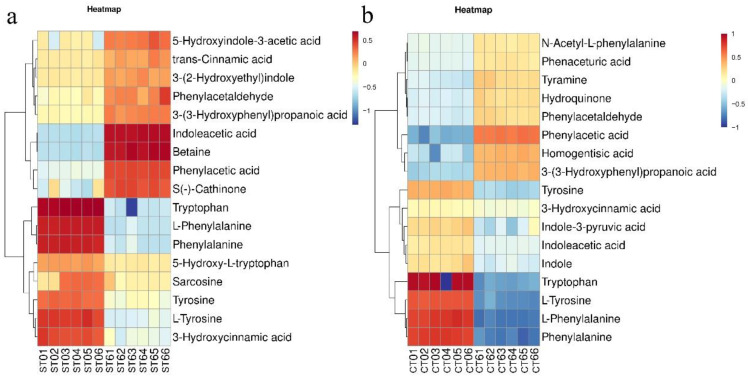
Analysis of different metabolites related to the amino acid metabolism pathways in the aqueous extraction group (**a**) and the alcohol extraction group (**b**) (ST—aqueous extraction group; CT—alcohol extraction group; the first number represents the fermentation day of the sample added; the second number represents the repeat group).

As shown in Figure 10, the intake of aqueous extract significantly upregulated 10 metabolites related to amino acid metabolic pathways. These metabolites, which included 5-hydroxyindole-3-acetic acid, 3-(2-hydroxyethyl) indole, indoleacetic acid, betaine, and eight related metabolites, were significantly downregulated, such as tryptophan, L-phenylalanine, phenylalanine, L-tyrosine, and tyrosine.

The intake of alcohol extract significantly increased nine metabolites related to amino acid metabolic pathways, including phenylacetic acid, tyramine, and homogentisic acid. In addition, the alcohol extract reduced the abundances of indoleacetic acid, indole, tryptophan, L-tyrosine, L-phenylalanine, phenylalanine, and other related metabolites.

### 3.12. Analysis of Metabolites Related to the Lipid Metabolism Pathways of Intestinal Microbiota

**Figure 11:**
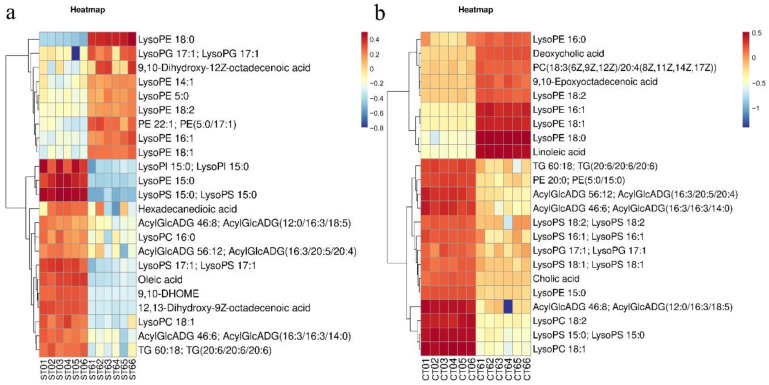
Analysis of different metabolites related to lipid metabolism pathways in the aqueous extraction group (**a**) and the alcohol extraction group (**b**) (ST—aqueous extraction group; CT—alcohol extraction group; the first number represents the fermentation day of the sample added; the second number represents the repeat group).

As shown in Figure 11, the intake of both aqueous and alcohol extracts resulted in significant changes in lysopids (LysoPC, LysoPE, LysoPG, LysoPI). Intake of aqueous extracts also downregulated hexadecanedioic acid, oleic acid, and acylGlcADG. However, the alcohol extract significantly upregulated deoxycholic acid, 9,10-epoxyoctadecenoic acid, and linoleic acid, and significantly downregulated cholic acid.

## 4. Discussion

In this study, through an in vitro digestion simulation system, it was found that the two extracts of *Cistanche deserticola* had different effects on intestinal microbiota, which showed differences in alpha diversity, composition, and metabolism of intestinal microbiota. Therefore, the changes in intestinal microbiota and metabolism were analyzed in depth. Then, the potential effects of the two extracts on the human body were evaluated scientifically.

Alpha diversity is an important component of biodiversity in ecology and a comprehensive index reflecting richness and evenness. The interventions of both extracts did not interfere with the alpha diversity of intestinal lumen microbiota, indicating that the intervention of samples would not disrupt the homeostasis of the intestinal microbiota. However, after PCA, both extracts had significant effects on the composition of the flora, and the effects were different. At the phylum level, the aqueous extract of *Cistanche deserticola* increased the ratio of *Firmicutes* to *Bacteroidetes*, while the alcohol extract decreased the ratio of *Firmicutes* to *Bacteroidetes*. The ratio of *Firmicutes* to *Bacteroidetes* has long been considered a significant biomarker of obesity [11]. The larger the ratio, the more likely the individual is to be obese. This indicates that long-term consumption of an aqueous extract of *Cistanche deserticola* is associated with an increased risk of obesity, while long-term consumption of an alcohol extract can reduce the risk of obesity. At the genus level, the intake of the two extracts can promote the growth of probiotic *Bifidobacterium* and promote intestinal health. In addition, intervention with the extracts can also increase the growth of *Prevotella* and reduce the number of *Bacteroides*. *Bacteroides* are associated with high fat and protein intakes, while *Prevotella* is associated with plant-rich diets, and they are mostly antagonistic. In this study, the extract of *Cistanche deserticola* was rich in carbohydrates, so it could significantly increase the relative abundance of *Prevotella*. Moreover, studies have shown that a higher ratio of *Prevotella*/*Bacteroides* has beneficial effects on host metabolism [12]. In addition, the aqueous extract group significantly inhibited *Phascolarctobacterium*, which is the producer of acetic and propionic acids and has a potential positive correlation with host positive emotions [13]. In the process of aqueous extract intake, the relative abundance of *Blautia* fluctuated, while there was no significant change in the alcohol extract group. According to the literature, *Blautia* can help clear away the gas in the intestine and use carbon dioxide, and hydrogen is converted into acetic acid salt [14]; these results indicate that the aqueous extract from the intervention will increase the risk of abdominal distension.

However, in the intestinal mucosa, the intervention of the two extracts resulted in different changes in the mucosal microbiota and intestinal lumen microbiota. First, both extracts promoted *Megasphaera* and *Enterobacter* in the mucosa, and Parabacteroides, *Ruminococcus 2*, *Escherichia-Shigella*, and *Collinsella* were inhibited. Some studies have reported that *Megasphaera* isolated from human feces has a variety of carbohydrate active enzymes, which can produce short-chain fatty acids and affect host health [15]. *Collinsella* has been reported to be associated with nonalcoholic steatohepatitis, which tends to promote inflammation at high abundance. Some genera of the *Ruminococcus* family have been proven to be proinflammatory, and the inhibition of *Ruminococcus* by the samples in this study may reduce the risk of intestinal inflammation [16]. In addition, the intervention of the aqueous extract caused fluctuations in *Olsenella* and *Lachnospiraceae*. As a potential probiotic, *Lachnospiraceae* is involved in the metabolism of a variety of carbohydrates, and its fluctuation leads to an increase or decrease in short-chain fatty acids, thus affecting intestinal homeostasis. An increase in the relative abundance of *Olsenella*, which is capable of producing SCFAs, protecting the intestinal barrier, or anti-inflammatory beneficial bacteria, can promote gut health.

The composition of intestinal gas can reflect the metabolism of intestinal microbiota to a certain extent. Ammonia, hydrogen sulfide, hydrogen, and methane are the main components of intestinal gas. In this study, the two groups of extracts easily produced methane after intervention, which was caused by the high contents of carbohydrates and amino acids in the samples and the fermentation of microorganisms [17]. According to Furnari et al., methane is closely related to constipation-type IBS and functional constipation [18]. Hydrogen, carbon dioxide, and SCFA are the main products of carbohydrate fermentation, and gas production is usually used as an indicator of dietary fiber fermentation in the colon. According to the above results, the aqueous extract could significantly increase the proportions of hydrogen and hydrogen sulfide compared with the alcohol extract. This indicated that the carbohydrates in the aqueous extract were easily fermented to produce hydrogen. Gut microbes use hydrogen to produce methane and hydrogen sulfide, and the presence of hydrogen also promotes the production of butyrate. A recent study found that hydrogen sulfide could reduce the sensitivity of intestinal mucosa to injury by regulating intestinal microbiota [19]. In addition, as an important component of microbial metabolites, SCFAs can also reflect the metabolic status of the intestinal microbiota to a certain extent. According to reports, SCFAs can provide 10% of the total energy of the human body, protect the intestinal mucosal barrier [20], regulate immunity and other effects [21], and reduce intestinal pH, which is conducive to the growth of probiotics, inhibits the growth of pathogenic bacteria or opportunistic bacteria, and is beneficial to intestinal health. In this study, both extracts of *Cistanche deserticola* promoted the production of SCFAs, especially butyric acid. Previous studies have shown that butyrate, which is mainly metabolized by Firmicutes, can inhibit acylase activity, regulate host gene expression, and play an important role in alleviating inflammation and reducing colon cancer [22]. It has been shown that butyrate can inhibit the high levels of proinflammatory factors secreted by macrophages by inhibiting NF-κB [23]. In conclusion, the intake of *Cistanche deserticola* extract can promote intestinal health.

To further understand the effects of the two extracts on the gut microbiota, untargeted metabolome analysis was performed in this study. Through the analysis of the different metabolites, it was found that the extract greatly affected the amino acid and lipid metabolism pathways of the intestinal microbiota. Indoles are mainly derived from the metabolism of tryptophan by intestinal microbiota, and indoleacetic acid is one of the important metabolites of intestinal microbiota. The microorganisms involved mainly include *Bacteroides*, *Bifidobacterium*, and *Clostridium* [24]. Studies have shown that indoleacetic acid can attenuate high-fat diet-induced hepatotoxicity in mice, which is believed to be related to insulin resistance, lipid metabolism, and oxidative and inflammatory stress [25]. Microbial metabolized products of tryptophan have also been shown to act on aryl hydrocarbon receptors (AHRs) found in intestinal immune cells to alter innate and adaptive immune responses in ligand-specific ways [18]. Su Xiaomin found that 3-indoleacetic acid (IAA) and LPS produced by intestinal microbiota (especially *Lactobacillus*) metabolism can promote IL-35+ B-cell (regulatory B cell) accumulation in adipose tissue by acting on progesterone X receptor (PXR) and Toll-like receptor 4 (TLR4), respectively. These effects inhibit the obesity induced by a high-fat diet in mice [26]. Betaine in fermentation solution is mainly derived from the aqueous extract of *Cistanche deserticola*. Some studies have suggested that betaine can improve the dysbiosis of intestinal microbiota induced by a high-fat diet (HFD) and increase short-chain fatty acid-producing strains in mice, which plays an important role in the prevention of obesity and metabolic syndrome [27]. However, the intervention of alcohol extract resulted in the downregulation of indoles and the upregulation of tyramine. Tyramine produced by bacteria can be converted to Simba amine by host tyramine β-hydroxylase [28]. Tyramine is also negatively correlated with a variety of inflammatory biomarkers and cardiometabolic risk factors [29]. In addition, the intake of alcohol extract is accompanied by a rise in high gentian acid, and there is a risk of alkaptonuria if it is not catabolized in a timely manner. In the analysis of lipid metabolic pathway-related metabolites, this study found that both kinds of extracts can cause lysophosphatide to change significantly. Lysophosphatide is a bioactive molecule that plays a role in a variety of various types of cells [30]. For example, LysoPC can change a variety of cell types in atherosclerosis and plays important roles in inflammatory diseases [31]. In addition, alcohol extract intake significantly increased deoxycholic acid, which may increase the intestinal risk [32].

In conclusion, the two kinds of extracts of *Cistanche deserticola* have significant effects on the intestinal microbiota and its metabolism.

## 5. Conclusions

In this study, the different effects of aqueous and alcohol extracts of *Cistanche deserticola* on gut microbiota and its metabolism were revealed by a CDMN in vitro gut simulation system. Intake of either extract did not decrease the alpha diversity of the gut microbiota. The intervention of aqueous extract can increase the F/B value, while the long-term intake of alcohol extract can decrease it. At the genus level, the two extracts inhibited *Bacteroides* in the intestinal lumen and *Parabacteroides* and *Ruminococcus* in the intestinal mucosa and promoted *Bifidobacterium* and *Prevotella* in the intestinal lumen and *Megasphaera* in the intestinal mucosa, while the aqueous extract also inhibited *Phascolarctobacterium*. All the extracts induced changes in intestinal gas and significantly promoted the production of methane, while the aqueous extract significantly increased the proportions of hydrogen and hydrogen sulfide. Both extracts still produced a significant increase in SCFAs in the gut, particularly butyrate production. In addition, the extract of *Cistanche deserticola* caused changes in various metabolic pathways of intestinal microbiota, especially amino acid- and lipid-related pathways. Among them, indoles were upregulated by aqueous extract and downregulated by alcohol extract. The two extracts also had significant effects on lysophospholipids. In conclusion, this study has guiding significance for different populations for consuming *Cistanche deserticola* and provides a theoretical basis for the food development of *Cistanche deserticola* to expand its application scope, which has important significance for the development of the *Cistanche deserticola* industry.

## Figures and Tables

**Table 1:** Main chemical constituents of extracts and their digested products.

	Type of Extract	Total Free Amino Acid Content (mg/mL)	Reducing Sugar Content (mg/mL)	Phenylethanol Glycosides Content (10^−2^ mg/mL)
Sample	aqueous extract	1.93 ± 0.34	116.18 ± 6.35	463.86 ± 3.44
	ethanol extract	2.04 ± 0.11	115.48 ± 10.48	216.20 ± 6.88
In vitro gastric digestion	aqueous extract	0.84 ± 0.03	14.39 ± 0.58	90.17 ± 7.18
	ethanol extract	0.78 ± 0.08	34.39 ± 0.24	80.00 ± 8.86
In vitro small intestinal digestion	aqueous extract	0.39 ± 0.01	7.69 ± 0.14	9.96 ± 2.28
	ethanol extract	0.37 ± 0.01	21.26 ± 0.31	15.33 ± 2.2

## Data Availability

Data is contained within the article.
